# Liposomal and Ethosomal Gels for the Topical Delivery of Anthralin: Preparation, Comparative Evaluation and Clinical Assessment in Psoriatic Patients

**DOI:** 10.3390/pharmaceutics12050446

**Published:** 2020-05-11

**Authors:** Dina Fathalla, Eman M. K. Youssef, Ghareb M. Soliman

**Affiliations:** 1 Department of Pharmaceutics, Faculty of Pharmacy, Assiut University, Assiut 71526, Egypt; dina.mohamed@pharm.aun.edu.eg; 2 Department of Dermatology, Venereology and Andrology, Faculty of Medicine, Assiut University, Assiut 71526, Egypt; emankyoussef@aun.edu.eg; 3 Department of Pharmaceutics, Faculty of Pharmacy, University of Tabuk, Tabuk 47512, Saudi Arabia

**Keywords:** psoriasis, anthralin, ethosomes, liposomes, topical drug delivery

## Abstract

To enhance anthralin efficacy against psoriasis and reduce its notorious side effects, it was loaded into various liposomal and ethosomal preparations. The nanocarriers were characterized for drug encapsulation efficiency, size, morphology and compatibility between various components. Optimum formulations were dispersed in various gel bases and drug release kinetics were studied. Clinical efficacy and safety of liposomal and ethosomal Pluronic^®^F-127 gels were evaluated in patients having psoriasis (clinicaltrials.gov identifier is NCT03348462). Safety was assessed by recording various adverse events. Drug encapsulation efficiency ≥97.2% and ≥77% were obtained for liposomes and ethosomes, respectively. Particle sizes of 116 to 199 nm and 146 to 381 nm were observed for liposomes and ethosomes, respectively. Fourier-Transform infrared (FT-IR) spectroscopy and differential scanning calorimetry (DSC) studies confirmed the absence of interaction between anthralin and various nanocarrier components. Tested gel bases showed excellent ability to sustain drug release. At baseline, the patients had a median Psoriasis Area and Severity Index (PASI) of 3.4 for liposomes and 3.6 for ethosomes without significant difference. After treatment, mean PASI change was −68.66% and −81.84% for liposomes and ethosomes, respectively with a significant difference in favor of ethosomes. No adverse effects were detected in both groups. Anthralin ethosomes could be considered as a potential treatment of psoriasis.

## 1. Introduction

Psoriasis is a chronic non-infectious autoimmune disease of the skin, joints or both characterized by relapsing episodes of inflammatory lesions and hyperkeratotic plaques [1,2,3]. It is the most prevalent autoimmune disease with a worldwide prevalence of 2–5% [4]. It affects people of all ages with a median age of symptoms onset at 28 years. More than a quarter of patients report symptoms beginning before the age of 18 years [5]. The exact cause of psoriasis is unknown, but both genetic and environmental factors such as trauma, drugs, infections, alcohol abuse, smoking and stress are known to contribute to the disease [6,7]. The most characteristic features of psoriasis include circumscribed, red, thickened plaques with an overlying silver-white scale [8]. This scale makes the disease highly visible and negatively impacts the patient’s lifestyle [7]. The visible nature of the disease leads to both physical and psychosocial effects on the patient including social disgrace, state of agony, distress and physical disability [2].

The management of mild to moderate psoriasis depends chiefly on topical therapies. Commercially available medications approved by the US Food and Drug Administration for this purpose include corticosteroids, vitamin D_3_ analogs, calcineurin inhibitors, retinoids, anthralin, and tar-based formulations [9,10]. Moderate to severe psoriasis management includes using the anti-tumor necrosis factor (TNF) α monoclonal antibodies adalimumab, etanercept and infliximab [11].

Anthralin (1,8-dihydroxy-9-anthrone), which was introduced over 80 years ago, has shown excellent efficacy in the management of psoriasis [12,13]. Anthralin has a molecular weight of 226.227 g/mol, poor aqueous solubility of less than 2 µg/mL and a Log *p* value of 1.99 [14,15]. Its mechanism of action involves inhibition of the proliferation of keratinocytes [16]. Further, accumulation of anthralin inside the mitochondria impairs energy supply to the cell, probably due to the free radicals resulting from oxidation of the drug [17]. Anthralin also interferes with the replication of DNA and slows down the extreme cell division that occurs in psoriatic plaques [18]. Although anthralin is remarkably effective in the management of psoriasis, its side effects are equally disturbing. Its use is messy as it stains the skin, clothing and any furniture that it may come in contact with. Further, anthralin has irritating, burning, brown discoloration and necrotizing effect on normal and diseased skin [14]. This troublesome profile has discouraged wide-spread use of the drug.

Several attempts have been made to incorporate anthralin into various drug delivery systems to overcome its shortcomings. For instance, a formulation containing microcrystalline monoglyceride-based microencapsulated anthralin has been developed (Micanol^®^) and showed less irritation and easy wash off from clothes [19]. Other approaches include drug incorporation into vesicular carriers (e.g., liposomes and niosomes), nanoemulsion, phospholipid microemulsion and nanocapsules [14,20,21,22]. The vesicles showed significantly higher permeation through mouse abdominal skin when compared to the cream base, in vitro [14]. Anthralin was also incorporated into polypropylene imine dendrimers [23]. The dendrimer-loaded drug showed significantly enhanced permeation rate constant and lower skin irritation. Anthralin loaded into lipid-core nanocapsules had better stability against UVA light-induced degradation and less toxicity compared with the drug solution [22]. 

Ethosomes are relatively new vesicular carriers composed mainly of phospholipids, ethanol and water. The intriguing features of ethosomes are due to their high ethanol content which facilitates their penetration through stratum corneum and target deep skin layers [2,24]. This is advantageous compared with conventional liposomes which have limited penetration through the skin and remain confined in the upper layer of the stratum corneum [25]. Compared to liposomes, ethosomes had greater retention of methotrexate into the skin for a longer period of time, suggesting a better therapeutic outcome [26]. It was shown that the quantity of tacrolimus remaining in the epidermis at the end of a 24-h experiment was significantly greater from the ethosomes than from commercial ointment. Further, in vivo topical application of ethosomal tacrolimus displayed efficient suppression of the allergic reactions compared to traditional liposomes and commercial ointment [27]. Zhang et al., showed that the transdermal flux and skin deposition of psoralen-loaded ethosomes were 3.5 and 2.15 times higher than those achieved using liposomes, respectively [28]. We also showed that ethosomes had ~6-fold higher voriconazole permeation through rat abdominal skin compared with the drug hydroalcoholic solution [29]. These results suggest that ethosomes could be a potentially effective delivery system to increase anthralin efficacy against psoriasis and limit its adverse effects. Careful literature review, however, shows no studies on ethosomal anthralin preparations. In addition, there is only one published clinical trial evaluating the effectiveness of anthralin-loaded liposomes [13]. 

In light of the above, the aim of the present study was to develop and evaluate an ethosomal gel preparation of anthralin and compare it with a liposomal gel preparation. The study reports, for the first time, a comparative clinical evaluation of anthralin-loaded ethosomes and liposomes in psoriatic patients. 

## 2. Materials and Methods

### 2.1. Materials

Anthralin was purchased from Professional Compounding Centers of America (Houston, TX, USA). Granular phosphatidylcholine from soybean (Phospholipon^®^ 90G, unsaturated diacyl-phosphatidylcholine content: 96.5%) (PL-90G) was a gift from Lipoid AG, Steinhausen, Switzerland. Cholesterol (CH), hydroxyethyl cellulose (HEC), hydroxypropyl methylcellulose (HPMC), Pluronic^®^ F-127 (PL-127), and dialysis membranes (MWCO 6–8 kDa) were purchased from Sigma Aldrich, St. Louis, MO, USA. Chloroform, ethanol, methanol, diethyl ether and all other chemicals were obtained from the United Company for Chem. Med. Prep., Cairo, Egypt. 

### 2.2. Preparation of Anthralin-Loaded Liposomes

Anthralin-loaded liposomes were prepared by the thin-film hydration method [30,31]. Different liposomal formulations were prepared using varied cholesterol/PL-90G and drug/lipid ratios (Table 1). Given amounts of anthralin, cholesterol and PL-90G were dissolved in a solvent mixture of chloroform and methanol (2:1, *v/v*) in a 250 mL round bottom flask. The organic solvent was evaporated under reduced pressure at 40 °C using a rotary evaporator (Büchi, type R 110, Flawil, Switzerland) resulting in the formation of a thin lipid film on the walls of the flask. To ensure complete removal of the residual organic solvents, the flask was left overnight in a vacuum desiccator. The thin lipid film was hydrated at 37 ± 1 °C using citrate buffer (10 mM, pH 3.3); 10 mL for each 100 mg total lipid. Subsequently, the obtained suspension was vortexed for about 2 min followed by incubation at room temperature for 2–3 h to allow complete hydration of the lipid film. The liposomal suspension was kept in the refrigerator to mature overnight (4 °C). The liposomes were ultrasonicated using a Cole-Parmer ultrasonic processor (model CPX 400, Cole-Parmer Instruments, Vernon Hills, IL, USA) for 2 min at 25% amplitude and frequency of 2 s ON and 2 s OFF. 

### 2.3. Preparation of Anthralin-Loaded Ethosomes

Anthralin-loaded ethosomes were prepared by the cold method as described previously with some modifications [29,32]. Table 2 shows the different components of ethosomes and their proportions. Anthralin (0.1%, *w/v*) and PL-90G (3–5%, *w/v*) were dissolved in the designated amount of absolute ethanol (35–45%, *v/v*) and stirred using a magnetic stirrer for 10 min until complete dissolution was obtained. Deionized water adjusted to pH 3.3 (QS to 100%) was slowly added using a syringe to the magnetically stirred ethanolic mixture and the preparation was stirred at 700 RPM for 5 min at room temperature. The mixture was ultrasonicated using a Cole-Parmer ultrasonic processor (model CPX 400, Cole-Parmer Instruments, Vernon Hills, IL, USA) for 2 min at 25% amplitude and frequency of 2 s ON and 2 s OFF. Blank ethosomes were prepared following the same procedures and used as a control. Blank and drug-loaded ethosomes were kept at 4 °C for further analysis. 

### 2.4. Characterization of Anthralin-Loaded Vesicles 

#### 2.4.1. Determination of Drug Encapsulation Efficiency 

Anthralin content of the liposomes and ethosomes was estimated by centrifuging a sample at 36,670× *g* and 4 °C for 30 min on a refrigerated centrifuge (Hettich, Germany). The clear supernatant was collected and an aliquot was properly diluted and its spectrophotometric absorbance was recorded at 349 nm. The obtained pellet was washed thrice with distilled water then lysed in methanol, sonicated for 10 min, properly diluted and the absorbance of an aliquot was measured at 349 nm. The anthralin concentration was calculated using a calibration curve (Y = 0.0401X + 0.0058, *R*^2^ = 0.9981). Drug encapsulation efficiency was calculated using the following equation:

(1) $$\mathrm{Encapsulation}\;\mathrm{efficiency}\;\left(\mathrm{weight}\;\%\right)=\frac{T-C}{T}\times 100,$$

where T is the total amount of drug in the supernatant and sediment, and C is the amount of drug in the supernatant [31]. Three different formulations were tested and the average ±SD was calculated.

#### 2.4.2. Thermal Analysis Studies

Thermal analysis of anthralin, PL-90G, cholesterol, their physical mixture, anthralin-loaded liposomes (L5), ethosome physical mixture and anthralin-loaded ethosomes (E4) was carried out using a differential scanning calorimeter (DSC, TA-60, Shimadzu, Kyoto, Japan). The calibration of the instrument was done using an indium standard. Samples (3−5 mg) were accurately weighed into DSC aluminum pans having a capacity of 50 μL and thickness of 0.1 mm. The loaded pans were press-sealed with an aluminum cover. Empty pans were used as a reference. The samples and reference were equilibrated to 25 °C and held at this temperature for 5 min then heated to 300 °C at a rate of 10 °C/min. The samples were purged in a stream of dry nitrogen flowing at a rate of 50 mL/min. The physical mixtures had the same composition as the liposomes L5 and ethosomes E4. 

#### 2.4.3. Fourier-Transform Infrared Spectroscopy (FT-IR) Studies

The studied samples were anthralin, PL-90G, cholesterol, their physical mixture, anthralin-loaded liposomes (L5), ethosome physical mixture and anthralin-loaded ethosomes (E4). Samples (3–5 mg) were mixed with potassium bromide (IR grade), compressed into disks under vacuum and analyzed on a JASCO FT-IR–4200 type A (JASCO Co., Tokyo, Japan). The scanning was done in the range of 4000–400 cm^−1^ at a resolution of 0.48–1.93 cm^−1^. 

#### 2.4.4. Measurement of Particle Size and Polydispersity Index 

These measurements were carried out using a Malvern Nano-ZetaSizer (Nano-ZS, Malvern Instruments, Worcestershire, UK) having a He-Ne laser operating at 633 nm and an avalanche photodiode detector. Different ethosomal and liposomal formulations (Table 1 and Table 2) were diluted 100-fold by distilled water and measured in triplicate at room temperature. The autocorrelation function was analyzed by a cumulant analysis method to calculate the Z-average particle size (hydrodynamic diameter) and the polydispersity index. 

#### 2.4.5. Transmission Electron Microscopy (TEM) Measurements 

Transmission electron microscopy (TEM) samples were prepared by adding 20 μL of the vesicle suspension onto a Formvar-coated 300 mesh grid stabilized with evaporated carbon film for 1 min. The excess sample was removed by a piece of filter paper. The samples were negatively stained by adding 20 μL of 2% aqueous uranyl acetate solution for a few seconds. The excess solution was removed by a piece of filter paper. The samples were allowed to dry overnight at room temperature and then observed by a TEM (JEOL 100 CX, Tokyo, Japan) operating at 80 KV.

### 2.5. Incorporation of Anthralin-Loaded Vesicles into Different Gel Bases

Anthralin-loaded liposomes (Formula L5) and ethosomes (Formula E4) were suspended in gel bases prepared from various polymers, namely Pluronic^®^ F-127 (PL-127) 20% *w/w*, hydroxyethyl cellulose (HEC) 2% *w/w* and hydroxypropyl methylcellulose (HPMC) 2% *w/w*. PL-127 gels were prepared by the cold method as previously described by Schmolka [33]. The given amount of PL-127 was dispersed in cold distilled water and the dispersion was kept in the fridge at 4 °C overnight until the mixture became a clear solution. HPMC and HEC gels were prepared by dispersing the required quantity of polymer in distilled water. The aqueous dispersion was allowed to hydrate for 4–5 h until a clear gel was obtained. The gel viscosity was measured using Brookfield DV + II model LV viscometer using spindle S-96 at 1.5 rpm and at 25 °C.

Drug-loaded liposomes (L5) and ethosomes (E4) were prepared as described above, centrifuged at 36,670× *g* and 4 °C for 30 min and the obtained pellets were mixed with given quantities of the gels so that final anthralin concentration in the gel was 1.0% *w/w*. The gels were vortexed until homogenous preparations were obtained. 

### 2.6. In Vitro Drug Release Studies 

Drug release studies were conducted using the dialysis bag method on samples of anthralin-loaded liposomal and ethosomal gels in comparison with the corresponding liposomal and ethosomal suspensions. A given weight of the gel was added to 1 mL of the release medium and transferred to a dialysis membrane (MWCO: 6–8 kDa). A similar volume of the drug solution in the release medium was similarly treated and used as a control. The dialysis membranes were placed into screw-capped glass tubes containing 25 mL of the release medium consisting of citrate buffer (10 mM, pH 3.3) containing 30% methanol and 2% sodium lauryl sulfate. The tubes were shaken at 50 RPM in a mechanical water bath shaker maintained at 37 °C. At predetermined time intervals, 3-mL samples were withdrawn from the release media and replaced by an equal volume of fresh buffer. Anthralin content of the samples was determined spectrophotometrically at 349 nm. Each experiment was run in triplicate and the mean cumulative percent drug released was plotted against time. 

### 2.7. Kinetic Analysis of Drug Release Data

In order to reveal the mechanism of anthralin release, its release data from various formulations were treated using various drug release kinetic models. Thus, the release data were fitted using zero-order, first-order, Higuchi model, Baker–Lonsdale model, Hixson–Crowell cube root equation and Korsmeyer–Peppas equation [34,35,36]. 

### 2.8. Ex Vivo Permeability Studies

These studies were carried out using rat abdominal skin according to previously published procedures [29,37]. The abdominal skin was obtained from Wistar male albino rats (6–8 weeks old) weighing 90–100 g. All animals were treated in accordance with the ethical guidelines approved by Faculty of Pharmacy, Assiut University (ethical approval number: S4–19, 1-9-2019), which follows the Guide for the Care and Use of Laboratory Animals, 8th Edition, National Academies Press, Washington, DC. The rats were sacrificed and the abdominal region was freed from hair by the aid of an electric clipper. The connective tissue, fat and/or subcutaneous tissue were carefully removed from the abdominal skin. The skin was carefully examined to exclude samples with irregularities, such as fine holes or crevices then washed with normal saline and dried between two filter papers. The skin was used directly without storage. The skin was mounted in a test tube open from both sides with the stratum corneum side facing upward. The receiver compartment contained 100 mL of citrate buffer (10 mM, pH 3.3) containing 30% methanol and 2% sodium lauryl sulfate and maintained at 37 °C. The diffusion area of the skin was 0.785 cm^2^. The donor compartment was loaded with 300 mg of anthralin-loaded liposomal PL gel (formulation L5) or anthralin-loaded ethosomal PL gel (formulation E4). Anthralin concentration in all the gel samples was 1% *w/w*. Anthralin solutions in either the receptor medium or ethanol/water pH 3.3 mixture (40:60, *v/v*) were used as controls. The receptor medium was magnetically stirred at 100 rpm. At various time intervals (1, 2, 3, 4, 5 and 6 h), 5-mL samples of the receptor medium were withdrawn and immediately replaced with fresh receptor medium. Anthralin concentration in the collected samples was measured spectrophotometrically at 349 nm. Cumulative anthralin amount permeated through the skin (µg·cm^−2^) was calculated for both preparations. At the end of the drug permeation studies, the amount of drug deposited in the skin was determined. For this purpose, any remaining formulation on the skin surface was wiped off and the skin was gently washed with water three times to remove any adhering formulation. The skin was cut into small pieces and kept in methanol for 24 h to extract the drug. The methanolic extract was filtered through 0.2 µm filter and used to determine drug concentration spectrophotometrically at 349 nm. All experiments were done in triplicate. The steady-state flux (*Js*, µg.cm^−2^·h^−1^) was taken as the slope of the linear regression line resulting from plotting the cumulative drug amount permeated (µg·cm^−2^) against time [38]. The apparent permeability coefficient (P_app_, cm·h^−1^) was calculated from Equation (2).

(2) $${\mathrm{Papp}}_{}=\frac{Js_{}}{C_{0}},$$

where *Js* is the steady-state flux and *C*_0_ is the initial anthralin concentration in the donor medium. 

### 2.9. Clinical Evaluation of Selected Anthralin Liposomal and Ethosomal Formulations

#### 2.9.1. Study Design

This prospective, randomized, controlled, single-blind clinical trial was performed at the Department of Dermatology, Venereology and Andrology in Assiut University Hospital, Assiut, Egypt. The study design was approved by the Institutional Ethics and Research Committee of the Faculty of Medicine, Assiut University (Approval No. 17300079, 4-10-2017). All patients were informed about the study procedure, risks, benefits, potential complications and side effects and were included in the study after signing their informed consent. The trial followed the revised recommendations of ClinicalTrials.gov for improving the quality of reporting RCTs (clinicaltrials.gov identifier is NCT03348462).

#### 2.9.2. Patients

Twenty patients (16 males and 4 females) with stable chronic plaque psoriasis participated in the study. This study was conducted on psoriatic in-patients and out-patients attending the Department of Dermatology, Venereology and Andrology, Assiut University hospital. Inclusion criteria were: 

1—Patients with mild to moderate stable chronic plaque psoriasis. 

2—Age of patients ranged from 11 to 58 years (median: 46 years). 

Whereas, the exclusion criteria were: 

1—Patients with guttate, severe psoriasis or other inflammatory skin diseases.

2—Patients who received any topical or systemic treatment for psoriasis one month before the start of the study.

3—Pregnant or lactating females.

Patients were randomly divided into two groups. Group I included 10 patients who were treated with a once-daily application of liposomal gel preparation of anthralin (formulation L5 suspended in PL-127 gel). Group II included 10 patients who were treated with a once-daily application of ethosomal gel preparation of anthralin (formulation E4 suspended in PL-127 gel). A detailed history (age, age at onset, sex, occupation, residence, marital status, duration of disease, precipitating and exacerbating factors, family history and previous treatment for psoriasis) was obtained from each patient. A thorough clinical dermatological examination was done and Psoriasis Area and Severity Index (PASI) scores were obtained for all patients. A skin biopsy from the psoriatic lesion using a 5 mm punch was done to all patients before and after completion of the treatment. 

#### 2.9.3. The Application Protocol 

Anthralin preparation was applied to the psoriatic plaque by a cotton-tipped applicator and left for one hour only followed by washing the treated lesion with water. This was done under the doctor’s and nurse supervision. This supervised treatment regimen was done daily to all the studied psoriatic patients admitted to the hospital and for the first 4 days for unadmitted psoriatic patients. At home, the patient had to apply the preparation according to the supplied instructions. The treatment of all patients lasted for 8 weeks. Patients who were admitted in the hospital were seen daily and others who used treatment at home were seen twice weekly up to 8 weeks.

#### 2.9.4. Evaluation

Patients were followed up clinically and by digital photography before and after treatment for up to 8 weeks. After the end of treatment, follow up was done monthly up to 3 months to detect recurrence. The lesion severity scores were calculated at each visit and registered. The scores were compared to the baseline value that was registered at the first visit. The effectiveness of the treatment was measured as the mean percentage improvement in PASI. Safety was assessed by recording adverse events like itching, burning sensation, staining of skin or clothes and erythema. Digital photography of the lesions was done to all patients before treatment and weekly up to 8 weeks, using a 14.1 mega-pixels Sony DSC-W390 digital camera (Sony, Tokyo, Japan) to assess changes in clinical appearance and evaluate the response of treatment. Patient satisfaction at the end of the study was evaluated by the patient’s self-assessment of the degree of improvement of psoriasis by a single question (satisfied or not?). 

Histopathological examination of psoriatic lesions was done before and after treatment for both groups. All biopsied tissues were fixed in 10% formalin, routinely processed and embedded into paraffin. Five-micron thick sections were cut from the paraffin blocks and then stained with hematoxylin and eosin for routine histopathological evaluation. The tissue sections were observed under light microscope (Olympus, CH20, Tokyo, Japan) for the detection of histopathological changes in both pre- and post-treatment specimens. 

### 2.10. Statistical Analysis

All the experiments were run in triplicate and the results were reported as mean ± SD. The obtained data were statistically analyzed using GraphPad Prism software version 5.0 (GraphPad Software Inc., La Jolla, CA, USA). Analysis of variance (ANOVA) with Tukey method as a post-hoc test and Student’s *t*-test were used to analyze the data. The differences were considered statistically significant at *p* < 0.05.

## 3. Results and Discussion 

### 3.1. Preparation and Evaluation of Anthralin-Loaded Liposomes 

#### 3.1.1. Effect of Drug/Lipid Ratio on Anthralin Encapsulation Efficiency 

Anthralin-loaded liposomes were prepared by the thin-film hydration method at different drug/lipid ratios (Table 1). This method is one of the simplest methods to prepare liposomes on a small scale in a research laboratory [39]. The hydration medium was chosen as a citrate buffer (10 mM, pH 3.3) due to its known stabilizing effect on anthralin [14]. PL-90G was selected as the main lipid component in this study due to its low hydrolysis rate in acidic media. Thus, there was only 3% PL-90G hydrolysis after incubation in simulated gastric fluid pH 3 for 2 h at 37 °C [40]. 

To study the effect of the drug/lipid ratio, the liposomes were prepared at constant cholesterol/PL-90G ratio (0.3:0.7) and various drug/lipid ratios ranging from 10% to 75%. Results in Table 1 show that increasing the drug/lipid ratio from 10 to 25% was accompanied by a significant increase in the drug encapsulation efficiency from 97.2 ± 0.4% to 99.2 ± 0.2% (*p* < 0.05). Further increase in the ratio to 50% and 75% resulted in a significant increase in the drug encapsulation efficiency in comparison with the 10% ratio (*p* < 0.05). However, the difference in the drug encapsulation efficiency between 25%, 50% and 75% was non-significant (*p* > 0.05). The high drug encapsulation efficiency observed for all formulations might be attributed to the hydrophobic nature of anthralin (water solubility ≤2 µg/mL), which facilitates its incorporation in the liposome lipid bilayers. Similar results were previously observed for other drug-loaded liposomes [31,41]. High drug encapsulation efficiency is advantageous for clinical applications since it maximizes the drug/lipid ratio, which in turn reduces the cost of liposome preparation and limits the lipid-induced side effects [42]. The liposomes were separated by an ultracentrifugation method which gives a possibility of precipitation of unencapsulated anthralin due to its limited solubility in aqueous media. The pellets were purified by washing with distilled water three times which might limit the presence of the free unencapsulated drug. However, another purification method such as size exclusion chromatography or mini-column centrifugation should be used to confirm these results [14]. 

#### 3.1.2. Effect of CH/PL-90G Ratio on Anthralin Encapsulation Efficiency

Next, we studied the effect of CH/PL-90G ratio on the drug encapsulation efficiency at a constant drug/lipid ratio of 10% (Table 1). The drug encapsulation efficiency significantly decreased with the increase in the cholesterol content of the liposomes (*p* < 0.05). Thus, drug encapsulation efficiency significantly decreased from 97.2 ± 0.4% to 94.8 ± 0.1% when the CH/PL-90G ratio increased from 0.3:0.7 to 0.4:0.6 (Table 1) (*p* < 0.05). A further increase in the CH/PL-90G ratio to 0.5:0.5 resulted in a further significant decrease in the drug encapsulation efficiency (*p* < 0.05). Our previous studies showed that the encapsulation of another hydrophobic drug, latanoprost was not affected by increasing cholesterol concentration in the liposomes [31]. Other studies indicated that the encapsulation of hydrophobic drugs into liposomes usually increased upon increasing cholesterol concentration only if the initial drug input is less than the maximum encapsulation for a given drug [42]. This might explain the reduced anthralin encapsulation efficiency at higher cholesterol concentrations since the used drug concentration resulted in a high encapsulation efficiency of 97.2 ± 0.4% (Table 1). It is also noteworthy that the added concentration of cholesterol was at the expense of PL-90G which might be another reason for the reduced drug encapsulation. 

### 3.2. Preparation and Evaluation of Anthralin-Loaded Ethosomes

#### 3.2.1. Effect of PL-90G Concentration on the Drug Encapsulation Efficiency 

Anthralin-loaded ethosomes were prepared by the cold method at various PL-90G, ethanol and water concentrations and their drug encapsulation efficiency, particle size and polydispersity index were measured (Table 2). Turning our attention first to the effect of PL-90G concentration on the drug encapsulation efficiency, data in Table 2 show that at any given ethanol concentration there was a general decrease in the drug encapsulation efficiency with the increase in PL-90G concentration. Not all the differences were significant, though. Thus, at an ethanol concentration of 35%, the decrease in the encapsulation efficiency was significant (*p* < 0.05) except for that between 3% and 4% PL-90G. At 40% ethanol, the decrease was significant (*p* < 0.05) except for that between 4% and 5% PL-90G whereas the decrease at 45% ethanol was non-significant. Our previous study, among others showed the opposite effect where increasing PL-90G concentration had a positive effect on the encapsulation efficiency of other hydrophobic drugs [29,43]. The reason behind this difference is not clear but might be related to the relatively high anthralin drug encapsulation achieved in this study (≥77%, Table 2) where adding more PL-90G to the vesicle bilayer might squeeze out some of the encapsulated drug leading to a slight decrease in the encapsulation efficiency. The increase in PL-90G concentration was also accompanied by a decrease in the drug/lipid ratio which might be another reason for the decreased drug encapsulation efficiency (Table 2). It is noteworthy that the obtained encapsulation efficiency observed in our previous study was relatively intermediate (22.2 ± 1.1% to 46.5 ± 2.1%), which allowed more drug to be encapsulated with the increase in PL-90G [29].

#### 3.2.2. Effect of Ethanol Concentration on the Drug Encapsulation Efficiency

As for ethanol concentration effect on the drug encapsulation efficiency, PL-90G concentration was kept constant and the ethanol concentration was varied from 35 to 45%. Data in Table 2 show that the ethanol concentration had no effect as indicated by the non-significant difference in the drug encapsulation efficiency (*p* > 0.05). Some previous studies showed that increasing ethanol concentration decreased the encapsulation efficiency of various drugs [29,43,44,45]. Other studies showed the opposite effect where higher ethanol concentrations led to higher drug encapsulation efficiency [46,47]. These contradictory effects of ethanol on the drug encapsulation efficiency might be related to properties of the encapsulated drug, the range of the obtained encapsulation efficiencies, as well as the preparation method. Anthralin encapsulation efficiency for ethosomes was generally lower than that of liposomes. Thus, the encapsulation efficiency ranged from 91.8 ± 0.7% to 99.7 ± 0.0% for the liposomes and from 77.3 ± 0.7% to 85.0 ± 0.6% for the ethosomes, respectively. This is probably attributed to the presence of ethanol in ethosomes. During ethosome separation and determination of encapsulation efficiency the medium contained 35–45% ethanol. Anthralin solubility in ethanol probably facilitated its leakage from the ethosomes leading to lower encapsulation efficiency compared with liposomes. Similar effects were previously observed for other drugs incorporated into liposomes and ethosomes [48,49]. 

### 3.3. Particle Size and Polydispersity Index of Liposome and Ethosome Preparations

Particle size and polydispersity index (PDI) are essential parameters for nanoparticle characterization due to their effect on nanoparticle safety, stability, efficacy and in vivo performance as drug delivery systems [50]. The liposome particle size was in the range of 116.1 ± 1.7 to 199.3 ± 4.2 nm (Table 1) and did not follow a specific pattern. It was affected by neither CH/PL-90G nor drug/lipid ratios. PDI for all the liposome formulations was in the range of 0.2 to 0.9. PDI values smaller than 0.05 represent highly monodispersed populations whereas values greater than 0.7 indicate very broad particle size distribution [50]. In the case of liposomes, PDI values equal to or smaller than 0.3 are deemed acceptable and indicate a homogenous population of phospholipid vesicles [50,51]. 

Similar to liposome formulations, there was no definite trend for the particle size of the ethosome formulations (Table 2). The ethosome particle size ranged from 145.9 ± 0.8 to 381.3 ± 3.2 nm. The particle size was affected by neither the PL-90G nor the ethanol content of the ethosomes. Except for formulations E9, the PDI of all the other formulation was less than 0.7 confirming their acceptable size distribution (Table 2). Ethosome particle size is generally smaller than that of liposome due to the presence of ethanol in the ethosomes [52]. Results in Table 1 and Table 2 show that anthralin ethosomes were generally bigger than liposomes. However, the lipid concentration in both vesicular systems was not the same making it hard to compare the size and draw a valid conclusion. 

It has been previously shown that drug delivery to deeper layers of skin requires liposomal particle size smaller than 600 nm. Vesicles larger than 600 nm tend to stay on the stratum corneum and may dry to form a lipid layer on the skin [50,53]. Vesicles smaller than 300 nm were able to deliver their payload to some extent into deeper layers of skin whereas maximum drug delivery to deeper skin layers required particle size smaller than 70 nm [54]. The particle size data shown in Table 1 and Table 2 indicate that most liposome and ethosome formulations had a particle size smaller than 300 nm confirming their potential to deliver anthralin into deeper skin layers. Based on the above results liposome formulation L5 and ethosome formulation E4 were selected for further studies. These formulations showed high drug encapsulation efficiency (99.5 ± 0.0% and 85.0 ± 0.6% for liposome L5 and ethosome E4, respectively) and relatively small particle size (116.1 ± 1.7 nm and 201.5 ± 6.9 nm for liposome L5 and ethosome E4, respectively).

### 3.4. TEM Measurements 

**Figure 1:**
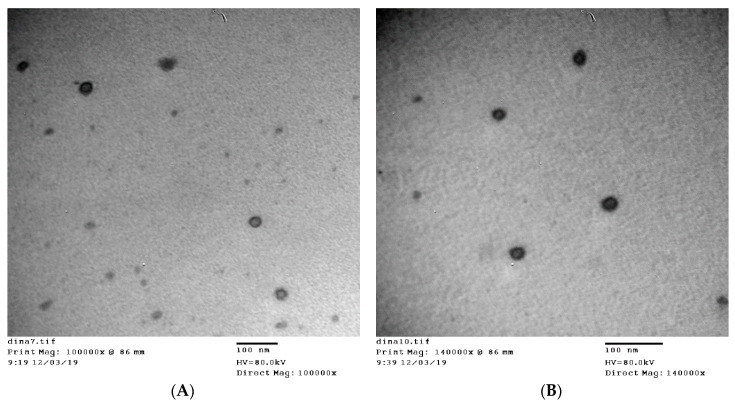
TEM photomicrographs of liposome formulation L5 (**A**) and ethosome formulation E4 (**B**).

Figure 1 shows TEM photomicrographs of liposome L5 (Figure 1A) and ethosome E4 (Figure 1B) formulations. Both formulations appear as discrete, round unilamellar vesicles with dark bilayer structure and light core. The size was 28.07 ± 4.02 nm for the liposomes and 21.63 ± 4.26 nm for the ethosomes. This particle size is significantly smaller than that observed by DLS measurements of the same formulations (Table 1 and Table 2). Previous results showed that nanoparticle size obtained by TEM measurements is usually smaller than that observed by DLS measurements [29,31]. DLS gives the hydrodynamic diameter of hydrated nanoparticles in solution, which is usually larger than the size of dried nanoparticles observed by TEM. 

### 3.5. Thermal Analysis Studies 

**Figure 2:**
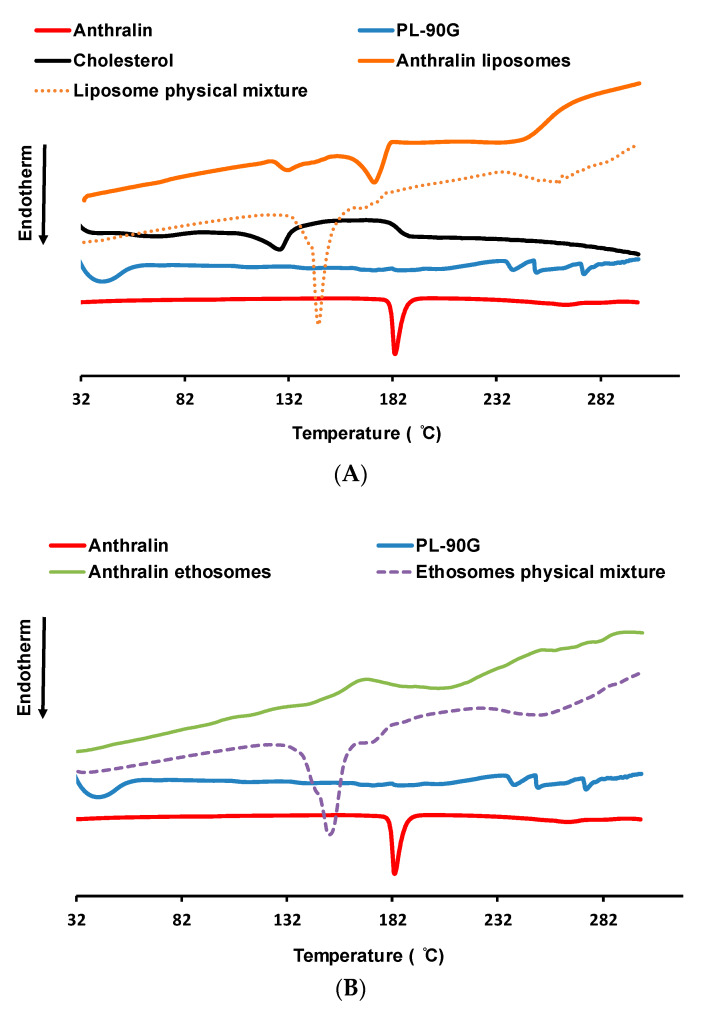
(**A**): Differential scanning calorimetry (DSC) thermograms of anthralin, PL-90G, cholesterol, liposome physical mixture and anthralin liposomes (L5). (**B**): DSC thermograms of anthralin, PL-90G, ethosome physical mixture, and anthralin ethosomes (E4).

Figure 2 shows DSC thermograms of liposomes L5 (Figure 2A) and ethosomes E4 (Figure 2B) in comparison with their various components. The DSC thermogram of anthralin shows a sharp endothermic peak centered at 183.1 °C due to drug melting [55]. PL-90G thermogram shows its phase transition temperature at 41.8 °C [56]. Cholesterol thermogram shows a melting endotherm at 127.3 °C [31]. The thermogram of the liposome physical mixture shows a sharp melting endotherm centered at 146.5 °C, which is probably due to anthralin melting endotherm. This downward shift in anthralin melting is probably due to lipid melting prior to the drug leading to drug solubilization and rapid melting [57]. Anthralin-loaded liposome thermogram shows endothermic peaks at 139.6 and 173.1 °C attributed, respectively to the melting of cholesterol and anthralin. Analogous to the liposome physical mixture, the ethosome physical mixture shows a downward shift of anthralin melting point to 152.29 °C (Figure 2B). The thermogram of anthralin-loaded ethosomes shows the disappearance of the drug melting peak (Figure 2B). 

Change of the position or disappearance of the drug and/or excipients thermal events is often suggestive of interactions between various components of a certain delivery system [31,57]. However, in the case of anthralin liposomes and ethosomes there were changes in the drug melting in the physical mixture, as well as in liposomes and ethosomes. This makes it hard to confirm the existence of interactions between anthralin and various liposome and ethosome components. 

### 3.6. FT-IR Studies 

**Figure 3:**
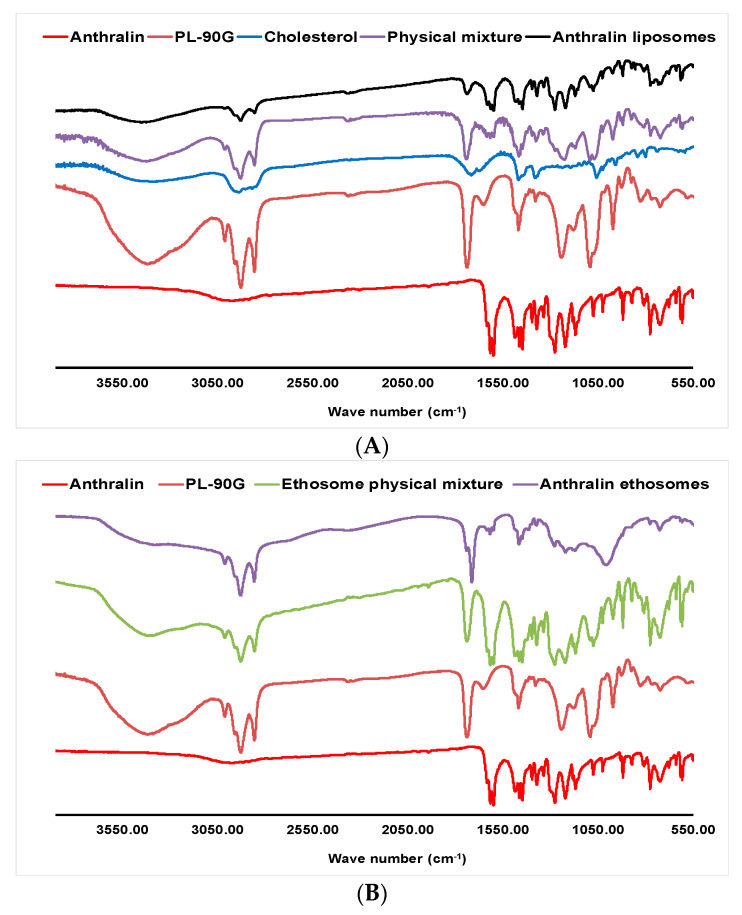
(**A**): Fourier-Transform infrared (FT-IR) spectra of anthralin, PL-90G, cholesterol, liposome physical mixture and anthralin liposomes (L5). (**B**): FT-IR spectra of anthralin, PL-90G, ethosome physical mixture, and anthralin ethosomes (E4).

Figure 3 shows the FT-IR spectra of liposomes L5 (Figure 3A) and ethosomes E4 (Figure 3B) in comparison with the spectra of their various components. The FT-IR spectrum of anthralin shows characteristic bands for OH bending and C=O stretching at 1633 cm^−1^, C–C stretching, OH bending and CH bending at 1615 cm^−1^, C–C stretching, C=O stretching and OH bending at 1598 cm^−1^ [58]. The spectrum of PL-90G shows C–H stretching bands at 2925 cm^–1^ and 2854 cm^−1^, a stretching band of ester carbonyl group at 1736 cm^−1^ and an ester C-O stretching band at 1240 cm^−1^ [31]. Cholesterol spectrum shows an absorption band at 3419 cm^−1^ due to O-H stretching. The bands in the region of 2800–3000 cm^−1^ are attributed to asymmetric and symmetric stretching vibrations of CH_2_ and CH_3_ groups [59]. The spectrum of the liposome physical mixture shows characteristic bands at 2926, 2854, 1740, 1635, 1616 and 1599 cm^−1^ due to the various absorption bands of its components. The spectrum of drug-loaded liposomes (L5) shows absorption bands at 2926, 2854, 1712, 1633, 1616 and 1598 cm^−1^. These bands appear at almost the same positions as those of the individual liposome components confirming that drug loading was not accompanied by interactions between various liposome components. 

Similar to the liposome physical mixture, the ethosome physical mixture shows absorption bands at wave numbers of 2925, 2854, 1737, 1615 and 1598 cm^−1^, which are due to the absorption bands of its components (Figure 3B). Ethosome E4 spectrum shows absorption bands at 2926, 2855, 1739, 1711, 1616 and 1599 cm^−1^. These bands show very little shift compared to those observed for the bands of the individual ethosome components. Therefore, no specific interactions took place between the drug and ethosome ingredients during ethosome preparation and drug loading. Taken together, the DSC and FT-IR studies suggest that anthralin loading into liposomes and ethosomes did not involve specific interactions between the drug and various nanocarrier components. 

### 3.7. In Vitro Drug Release Studies 

**Figure 4:**
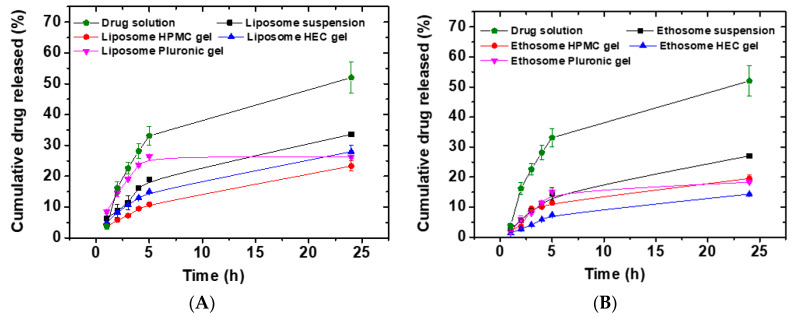
In vitro cumulative percent anthralin released as a function of time from anthralin-loaded liposomes (L5) and anthralin-loaded liposomes suspended into various gel bases (**A**) and from anthralin-loaded ethosomes (E4) and anthralin-loaded ethosomes suspended into various gel bases (**B**).

Aqueous buffers are not suitable as release media for anthralin due to its limited aqueous solubility (less than 2 µg/mL) [14]. Previous reports used a mixture of citrate buffer pH 3.3 containing 20% methanol and 1% Cremophor^®^ RH 40, which was found to be a satisfactory release medium for anthralin [14]. In our case to maintain sink conditions a medium of citrate buffer pH 3.3 containing 30% methanol and 1% sodium lauryl sulfate was used. Figure 4 shows the in vitro release profiles of anthralin from various liposomal (Figure 4A) and ethosomal formulations (Figure 4B). Drug solution in the release medium was used as a control to make sure that the dialysis membrane was not a barrier for drug diffusion. After 24 h, this solution showed a cumulative percent drug released of 52.01 ± 5.07%, which was significantly higher than that released from any of the tested formulations after the same time (*p* < 0.05). This confirms that the dialysis membrane did not impede the diffusion of released anthralin. Among various liposomal formulations, anthralin liposomes dispersed in PL-127 gel had the fastest drug release rate up to 5 h. The reason for this fast anthralin release is not clear but might be related to the ability of PL-127 to partly solubilize hydrophobic drugs [60,61]. After 24 h, the cumulative percent drug released from liposome suspension (33.6 ± 0.7%) was significantly higher than that released from all other liposomal preparations (*p* < 0.05). Incorporation of the liposomes into various gel bases (HEC, PL-127 and HPMC) resulted in significant reduction in the cumulative percent of anthralin released after 24 h (*p* < 0.05). The release rate after 24 h followed this order: liposome suspension > HEC gel > PL-127 gel > HPMC gel. Thus, the cumulative percent of anthralin released from liposome suspension, and liposomes dispersed in HEC, PL-127 and HPMC gels were, respectively 33.6 ± 0.7%, 27.9 ± 2.1%, 26.3 ± 0.6% and 23.3 ± 1.7%. These results confirm the ability of the tested formulations to sustain the drug release rate. All the differences were statistically significant (*p* < 0.05) except those between the PL-127 gel and either HPMC or HEC gel. 

Likewise, ethosome suspension had the fastest drug release rate compared to other ethosomal preparations. Thus, the ethosome suspension had a cumulative percent anthralin released after 24 h of 27.1 ± 0.4% (Figure 4B). The cumulative percent drug released after 24 h followed this order: ethosome suspension > ethosome HPMC gel > ethosome PL-127 gel > ethosome HEC gel. The differences were statistically significant (*p* < 0.05) except HPMC vs. PL-127 gel. Other reports showed similar findings where the dispersion of vesicular carriers into various gel bases resulted in reduced drug release rates [62,63]. It is believed that drug release from vesicles suspended in gel bases occurs through two steps; drug release from the vesicles, which act as a drug reservoir followed by diffusion through the gel network [64]. The presence of the gel network results in an increased diffusion path and subsequent reduction in drug release rate. Gel viscosity was reported to have an important influence on the drug release rate where increased viscosity was associated with a diminished drug release rate [65]. The viscosities of PL-127, HPMC and HEC gels were, respectively 720 ± 9.1, 100 ± 5.1 and 40 ± 2.1 Pa·s. Accordingly, it was expected that anthralin release rate should follow this order: HEC > HPMC > PL-127. Looking at the release rates from these gels, it seems that the gel viscosity had no influence on the drug release rate. This was unexpected but it is presumably attributed to the different nature of the polymers used in these gels. This causes a lot of differences that affect the drug release rate such as different gel dissolution rates and different specific interactions between the drug and polymers in the gel. Therefore, gel viscosity effect on drug release rate should be carefully compared within gels made from the same polymer [65]. 

When comparing the cumulative % of anthralin released from liposomes and ethosomes one notices that the release rate was generally slower for ethosomes. The opposite was expected since ethosomes contain ethanol which might solubilize the drug and facilitate its release from the vesicles. However, the volume of ethanol in the vesicles particularly after separation and dispersion in gel bases might be very small compared to the volume of the aqueous release medium thus, limiting its effect on the release process. In addition, the concentration of PL-90G in ethosomes was higher than that in liposomes, which might facilitate hydrophobic interactions with the drug leading to slower release. 

Liposomes (L5) and ethosomes (E4) dispersed in PL-127 gel showed an excellent ability to sustain the drug release where the cumulative percent of anthralin released after 24 h was, respectively 26.3 ± 0.6% and 18.3 ± 0.1%. PL-127 is a water-soluble nonionic triblock copolymer of poly(ethylene oxide) and poly(propylene oxide) with amphiphilic properties. It found several applications in the solubilisation and stabilization of various hydrophobic drugs [60]. In addition, PL-127 is well-known for its mucoadhesion, thermosensitivity and biocompatibility properties making it an excellent topical drug delivery carrier [31]. Accordingly, liposomes (L5) and ethosomes (E4) dispersed in PL-127 gel were selected for further studies. 

### 3.8. Drug Release Kinetics 

Table 3 shows the results of the kinetic analysis of anthralin release data using various mathematical models. The model having the highest correlation coefficient (*R^2^*) was chosen as the one best describing the drug release mechanism. All the investigated formulations (except Liposome PL-127 gel) had the highest correlation coefficient for the Baker-Lonsdale model. This model is used to describe drug release from spherical matrices with an emphasis on nanoparticle shape in controlling the drug release process [66]. Our previous studies showed that the release of another hydrophobic drug, spironolactone from liposomes and liposomes dispersed in methylcellulose gel was best described by the Baker-Lonsdale model [57]. Liposome PL-127 gel had the highest *R^2^* for the Higuchi model indicating that the drug release process was controlled by diffusion. This is in agreement with previous reports [67,68]. The correlation coefficients obtained for the Korsmeyer–Peppas equation were also quite high (0.8222 to 0.9934) (Table 3). Most of the values of the release exponent (n) were in the range of 0.43 < n < 0.85, which corresponds to anomalous (non-Fickian) transport [69]. 

### 3.9. Ex Vivo Permeability Studies 

**Figure 5:**
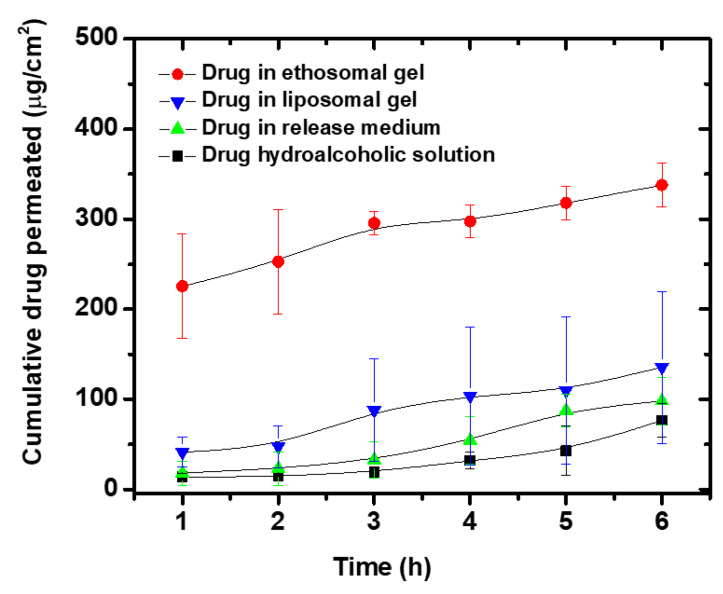
Ex vivo permeation profiles of anthralin from different preparations through rat abdominal skin.

Rat abdominal skin was used to test the potential of selected liposomal (L5 dispersed in PL-127 gel) and ethosomal (E4 dispersed in PL-127 gel) formulations to enhance anthralin permeation through the skin. Figure 5 shows the cumulative amount of anthralin permeated through the skin from various preparations as a function of time. Drug hydroalcoholic solution and drug solution in receptor medium, used as controls had slow permeation where after 6 h the amounts permeated were, respectively 76.67 ± 19.06 and 98.06 ± 26.74 µg·cm^−2^ (Table 4). Anthralin permeation from the liposomal gel was higher compared with these controls, though the differences were non-significant (*p* > 0.05). In contrast, anthralin permeation from the ethosomal gel was significantly higher than that from any of the other tested preparations (*p* < 0.05). Data in Table 4 show that the cumulative drug amount permeated from the ethosomal gel was around 2.5-, 3.5- and 4.5-fold higher than that from the liposomal gel, drug solution in receptor medium and drug hydroalcoholic solution, respectively (significant difference, *p* < 0.05). The steady state flux and apparent permeability coefficient were also higher for the ethosomal gel, yet the differences were non-significant (*p* > 0.05) (Table 4). Enhanced skin permeability of ethosomes has been previously observed in several other studies and was attributed to the presence of high ethanol concentration in the ethosomes [28,70,71]. Ethanol has the ability to increase flexibility and fluidity of the vesicles allowing them to deform and penetrate through skin pores that are smaller than their own size. However, ethanol is not the only factor operating since anthralin ethosomal gel had significantly higher drug permeation compared with the drug hydroalcoholic solution (Table 4, Figure 5). This is in agreement with previous reports which showed that ethosomes had higher drug permeation through skin compared to the drug hydroalcoholic solution [25,29]. Based on these observations, it was suggested that the observed enhanced drug permeation for ethosomes is dependent on several factors including lipid-softening effect of ethanol, as well as the interaction of ethanol and ethosome lipids with the stratum corneum lipids. This interaction might influence the bilayer structure of the stratum corneum [72]. Together, these effects might act in synergy resulting in enhanced penetration of the drug molecules across the stratum corneum and eventually better drug permeation [25].

The anthralin amount deposited in the skin after 6 h was higher for the liposomes compared with the ethosomes and the controls (Table 4). The difference was significant when compared with the drug hydroalcoholic solution or solution in the release medium but non-significant when compared with the ethosomes (*p* > 0.05). This higher skin deposition from liposomes is possibly due to drug deposition in the upper skin layers only, whereas ethosome fluidity and flexibility allowed them to penetrate deeper into various skin layers resulting in higher permeation. This is in agreement with other reports which confirmed that conventional liposomes deliver their cargo mostly to the upper layers of the startum corneum due to their limited penetration through the skin [25,72].

### 3.10. Clinical Evaluation of Selected Anthralin Liposomal and Ethosomal Formulations

In this prospective observational study, the effectiveness and safety of short-contact anthralin-loaded nanocarrier treatment in patients with psoriasis were demonstrated. Twenty patients participated in this study. Their mean age ±SD was 36.3 ± 16.3 and 43.1 ± 15.1 years for groups I and II, respectively (Table 5). Ten patients received anthralin-loaded liposomal PL-127 gel preparation (group I) and the other ten patients received anthralin-loaded ethosomal PL-127 gel preparation (group II).

The demographic characteristics did not differ significantly between the groups and within the same group in all parameters (Table 5). At baseline, the patients had an overall median PASI of 3.4 for group I, and 3.6 for group II without significant difference (*p* >0.05). After the end of the treatment, group I had a mean significant change in PASI score of −68.66%, while that of group II was −81.84%. These results are consistent with percentages reported in a randomized controlled trial involving short-contact anthralin therapy in adults, which demonstrated a PASI change of –63.3% [73]. The change in PASI for group II (ethosomes) was significantly higher than that of group I (liposomes) (*p* < 0.05). The mean treatment duration needed for improvement was found to be 21.7 days for Group I and 14.8 days for Group II with a significant difference between both groups (*p* < 0.05). Moreover, patient satisfaction for group II was significantly better than that of group I (*p* < 0.05) (Table 5). The maximum therapeutic effect of both groups was achieved just below the level of irritation of the surrounding uninvolved skin. This confirms that the prepared nanocarriers were able to abate the notorious anthralin side effects [74]. Anthralin staining is a commonly reported limitation [18,75]. 

It is worth mentioning that neither of the two tested formulations had adverse effects on the patients and none of them reported unwanted staining of their skin and clothes. In addition, the short period of treatment and the ease of application had increased patient compliance and adherence to treatment. Together, these findings highlight the potential of these preparations for clinical application.

**Figure 6:**
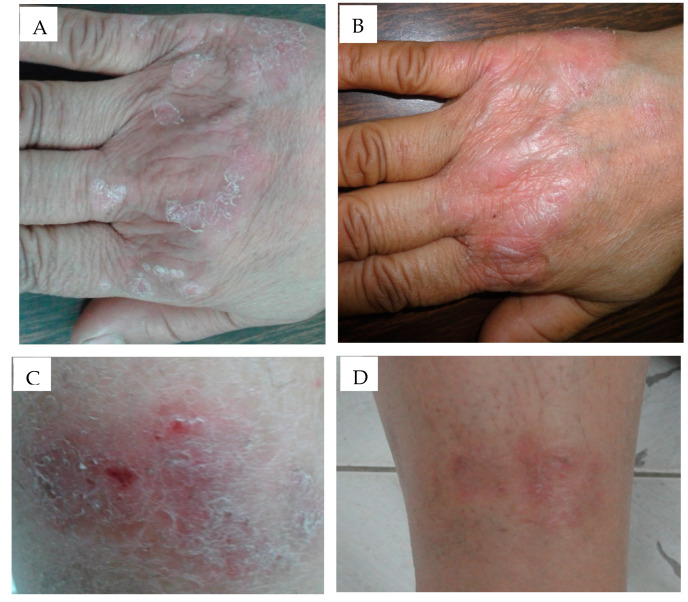
(**A**) A 44-year old male patient with a psoriasis vulgaris lesion on the dorsum of the right hand before treatment. (**B**) The same patient after 3 weeks of treatment with anthralin liposomal gel preparation showing marked improvement (Group I). (**C**) A 50-year old female patient with psoriasis vulgaris on the extensor surface of the right leg before treatment. (**D**) The same patient showing a marked improvement after 3 weeks of treatment with anthralin ethosomal gel preparation (Group II).

Digital photographs were taken for the patient before and after treatment with anthralin-loaded liposomal and ethosomal gel preparations in order to assess the degree of response to the therapy (Figure 6). Both groups showed marked improvement in psoriasis signs after 3 weeks of treatment.

**Figure 7:**
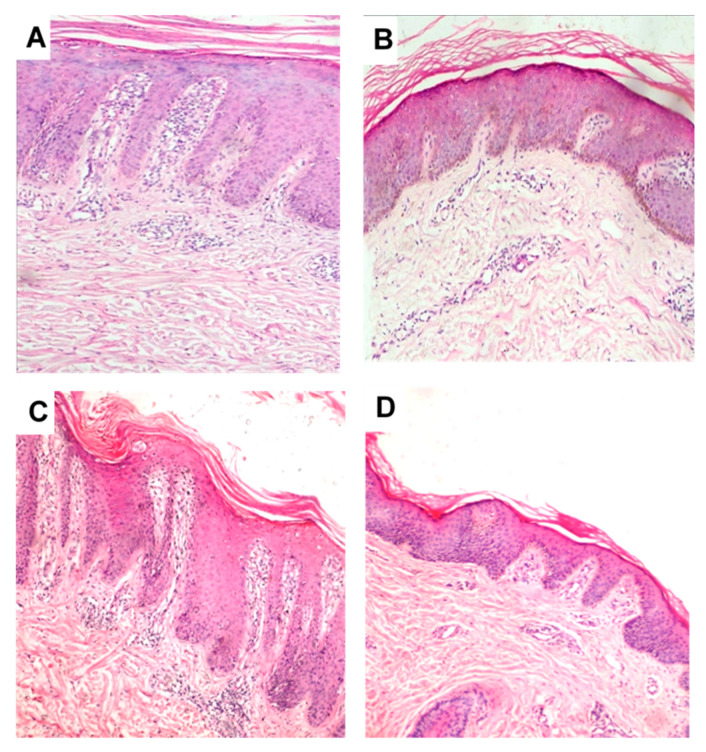
Histopathological manifestation of psoriasis vulgaris. (**A**) and (**B**) before and after treatment with anthralin liposomal gel preparation, respectively (Group I). (**C**) and (**D**) before and after treatment with anthralin ethosomal gel preparation, respectively (Group II). Magnification (10×, hematoxylin and eosin stain).

Histopathological examination of psoriatic lesions was done before and after treatment for both groups. The pretreatment sample of group I (Figure 7A) shows acanthosis, parakeratosis, with downward elongation of rete ridges (a comb-like structure), thin granular cell layer, suprapapillary thinning and prominent dermal capillaries. Mixed dermal infiltrate of lymphocytes, macrophages and neutrophils could also be seen. Following treatment with anthralin liposomal gel preparation (Figure 7B) there was a significant decrease in the findings described above illustrating marked improvement of the disease. Similar findings were observed for the samples obtained from group II before (Figure 7C) and after treatment (Figure 7D).

This study is the first to compare the effectiveness and safety of short-contact anthralin treatment application between two liposomal and ethosomal preparations. Obviously, it was found that the two nanocarrier preparations had good effectiveness and produced excellent therapeutic outcomes against psoriasis. There were significant differences in effectiveness, safety and improvement in patient quality of life between the two groups in favor of the ethosomal gel preparation (Table 5). Previous studies showed that anthralin liposomal preparations had equivalent clinical efficacy and lower irritation and staining compared with commercially available anthralin ointment. It is noteworthy that this effect was obtained at 56% lower drug concentration compared with anthralin ointment confirming the ability of liposomes to enhance drug efficacy [13]. It was assumed that this enhanced effect was due to the liposome nanosize and targeted drug delivery in a sustained-release fashion. In our study, the ethosomal preparation was more effective than that of liposomes, which is presumably due to the higher ethanol content of the ethosomes [76]. This gives ethosomes more fluidity and superior ability to penetrate through the skin and deliver their cargo to deeper skin layers, which in turn results in better therapeutic outcome [29,77,78].

A limitation of this study is the relatively small treatment groups and the absence of conventional anthralin preparation as a control. The number of patients used in this study is consistent with that reported in the only published clinical trial on anthralin liposomes (23 patients) [13]. A conventional anthralin preparation such as cream or ointment was not used as a control due to its disturbing side effects such as burning sensation and staining of the skin and clothes of the patients in addition to poor improvement of psoriasis lesions. Our previous studies (unpublished data) showed that none of the 12 patients involved continued using a commercial anthralin cream for more than 10 days because of marked irritation and black discoloration of their diseased and peri-lesional skin. However, considering the fact that data and evidence of treatment in patients with psoriasis using anthralin nanoparticles are very limited, we assume that these limitations are acceptable and conclusions could be drawn from the obtained results.

## 4. Conclusions

Anthralin-loaded ethosomal preparations were efficiently prepared by a simple procedure and compared with liposomes as a means to improve the safety and efficacy of anthralin. Both liposomal and ethosomal preparations had a particle size in the nanometer range. Optimum liposomal and ethosomal formulations in terms of particle size and drug encapsulation efficiency were incorporated into various gel bases to facilitate administration to the patients. Ex vivo permeability studies showed that anthralin ethosomal gel had significantly higher permeation through rat abdominal skin when compared with the drug liposomal gel. Clinical evaluation of anthralin liposomal and ethosomal preparations in psoriatic patients revealed that both preparations were able to minimize drug side effects. The observed PASI score indicated that ethosomes were much more effective than liposomes. Taken together, these results revealed that the merits of the developed anthralin ethosomal gel as an effective and safe treatment in psoriatic patients justify their potential in strengthening the efficacy and safety of the drug.

## Figures and Tables

**Table 1:** Composition and properties of different anthralin liposomal formulations.

Code	Drug/Lipid (wt%)	CH/PL-90G Ratio ^a^	Particle Size (nm) ^b^	PDI ^c^	Encapsulation Efficiency (%) ^d^
L1	10	0.3:0.7	198.9 ± 2.2	0.4 ± 0.01	97.2 ± 0.4
L2	10	0.4:0.6	143.1 ± 5.7	0.9 ± 0.01	94.8 ± 0.1
L3	10	0.5:0.5	231.5 ± 0.7	0.5 ± 0.01	91.8 ± 0.7
L4	25	0.3:0.7	199.3 ± 4.2	0.7 ± 0.01	99.2 ± 0.2
L5	50	0.3:0.7	116.1 ± 1.7	0.2 ± 0.01	99.5 ± 0.0
L6	75	0.3:0.7	187.9 ± 4.6	0.3 ± 0.00	99.7 ± 0.0

Each formulation contained 100 mg of total lipid (PL-90G + CH). ^a^ Cholesterol/PL-90G (CH/PL-90G) ratio, *w/w*. For instance, the ratio 0.3:0.7 contained 30 and 70 mg of CH and PL-90G, respectively in 10 mL citrate buffer (10 mM, pH 3.3). ^b^ Z-average particle size. ^c^ Polydispersity index. ^d^ Percent encapsulation efficiency, calculated from Equation (1). All the data are presented as the mean of three different measurements ± SD.

**Table 2:** Composition and properties of different anthralin ethosomal formulations.

Code	Drug/Lipid (wt%)	PL-90G (%, *w/v*)	Ethanol (%, *v/v*)	Particle Size (nm) ^a^	PDI ^b^	Encapsulation Efficiency (%) ^c^
E1	3.3	3	35	381.3 ± 3.2	0.1 ± 0.00	84.1 ± 3.1
E2	2.5	4	35	320.5 ± 8.6	0.4 ± 0.01	81.3 ± 1.1
E3	2	5	35	145.9 ± 0.8	0.3 ± 0.00	77.3 ± 0.7
E4	3.3	3	40	201.5 ± 6.9	0.4 ± 0.00	85.0 ± 0.6
E5	2.5	4	40	199.6 ± 5.7	0.4 ± 0.01	81.3 ± 0.7
E6	2	5	40	233.5 ± 2.2	0.3 ± 0.01	80.8 ± 2.0
E7	3.3	3	45	234.1 ± 6.9	0.7 ± 0.04	81.4 ± 0.3
E8	2.5	4	45	225.5 ± 3.6	0.5 ± 0.00	79.2 ± 0.9
E9	2	5	45	358.3 ± 5.8	1.0 ± 0.00	77.4 ± 3.5

Anthralin concentration was 0.1%, *w/v* in all the formulations (100 mg per 100 mL of formulation). Distilled water was QS up to 100%, *v/v* in all the formulations. PL-90G amounts were 3, 4 and 5 g per 100 mL of formulation for the 3%, 4% and 5%, *w/v* formulations, respectively. ^a^ Z-average particle size. ^b^ Polydispersity index. ^c^ Percent encapsulation efficiency, calculated from Equation (1). All the data are presented as the mean of three different measurements ± SD.

**Table 3:** Kinetic analysis of anthralin release data.

Formulation	Zeroth-Order	First-Order	Higuchi Model	Hixson Crowell Model	Baker–Lonsdale Model	Korsemeyer–Peppas Equation	
	*R* ^2^	*R* ^2^	*R* ^2^	*R* ^2^	*R* ^2^	*R* ^2^	n
Liposome	0.9455	−0.9604	0.9813	0.9557	0.9926	0.9779	0.54
Liposome HPMC gel	0.9766	−0.9827	0.9976	0.9808	0.9996	0.9934	0.59
Liposome HEC gel	0.9585	−0.9694	0.9913	0.9660	0.9973	0.9859	0.53
Liposome PL-127 gel	0.5847	−0.5939	0.7044	0.5909	0.6262	0.8222	0.34
Ethosome	0.9483	−0.9607	0.9843	0.9568	0.9954	0.9637	0.71
Ethosome HPMC gel	0.8830	−0.8982	0.9404	0.8933	0.9794	0.9115	0.69
Ethosome HEC gel	0.9504	−0.9566	0.9853	0.9545	0.9958	0.9680	0.73
Ethosome PL-127 gel	0.7717	−0.7852	0.8593	0.7808	0.8712	0.8608	0.69

**Table 4:** Ex vivo permeation parameters of anthralin from different formulations through rat abdominal skin.

Parameter	*Q* ^a^	*Js* ^b^	*P*_app_ × 10^3 c^	*Q* (skin) ^d^
Drug solution in receptor medium	98.06 ± 26.74	17.58 ± 3.88	14.56 ± 3.74	13.0 ± 4.9
Liposome PL-127 gel	135.7 ± 84.3	19.3 ± 15.8	17.4 ± 14.2	252.2 ± 106.9
Drug hydroalcoholicsolution	76.67 ± 19.06	11.79 ± 5.26	10.83 ± 4.84	31.4 ± 3.3
Ethosome PL-127 gel	337.6 ± 24.1	21.65 ± 8.0	19.9 ± 7.4	112.8 ± 21.9

^a^ Cumulative amount of anthralin permeated (µg·cm^−2^) after 6 h. ^b^ Flux (permeation rate constant) at steady state (µg·cm^−2^·h^−1^), obtained from the slope of the regression line after plotting the amount of anthralin permeated vs. time. ^c^ Apparent permeability coefficient (cm·h^−1^) calculated from Equation (2). ^d^ Amount deposited in the skin after 6 h (µg).

**Table 5:** Demographic information for the patients participating in the clinical evaluation of anthralin-ethosomal and liposomal preparations.

Parameter	Liposomal Preparations	Ethosomal Preparations	*p* Value
Number of patients	10	10	
Age (years), mean ± SD (range)	36.3 ± 16.3 (11–58)	43.1 ± 15.1 (13–55)	0.34
Sex Males/Females, n (%)	9/1 (90/10)	7/3 (70/30)	0.28
Residence%			
Rural	40%	20%	
Urban	60%	80%	
Occupation %			
Working	90%	70%	
Not working	10%	30%	
Family history			
Positive	30%	0%	
Negative	70%	100%	
Age at onset (years), mean ± SD (range)	31.9 ± 14.3 (10.9–53)	37.3 ± 12.8 (12.6–48)	0.39
Duration of psoriasis (months), mean ± SD (range)	52.8 ± 67.4 (3–228)	70 ± 62.7 (6–204)	0.56
Provocation factors %			
Stress	60%	40%	
Sun exposure	0%	10%	
Trauma including itching	60%	70%	
Infection	10%	0%	
Chemicals like detergent	10%	0%	
Effectiveness of preparations			
Baseline PASI, median (IQR)	3.4 (1.2–3.6)	3.6 (1.2–4)	0.53
End of treatment PASI, median (IQR)	0.6 (0.2–1.2)	0.4 (0.2–1.2)	0.59
Percentage change in PASI (95%CI)	−68.66 (−77.44 to −59.88)	−81.84 (−87.02 to −76.67)	0.009 *
Short contact therapy			
Duration of treatment needed for improvement (days), mean ± SD	21.7 ± 2.2	14.8 ± 4.8	0.0006 *
Adverse effects	No side effects	No side effects	
Patient satisfaction			
Very satisfied (%)	30%	80%	
Satisfied (%)	70%	20%	
Recurrence after end of treatment up to 3 months			
No recurrence	70%	80%	
Recurrence	30% after 1 month	20% after 2 month	

PASI, Psoriasis Area and Severity Index; IQR, interquartile range; CI, confidence interval; * Significant *p*- value.
